# Management of Chronic Diseases in Sub-Saharan Africa: Cross-Fertilisation between HIV/AIDS and Diabetes Care

**DOI:** 10.1155/2012/349312

**Published:** 2012-10-31

**Authors:** Josefien van Olmen, François Schellevis, Wim Van Damme, Guy Kegels, Freya Rasschaert

**Affiliations:** ^1^Department of Public Health, Institute of Tropical Medicine, Nationalestraat 155, 2000 Antwerp, Belgium; ^2^Netherlands Institute for Health Services Research (NIVEL), Otterstraat 118-124, 3513 CR Utrecht, The Netherlands; ^3^Department of General Practice and Elderly Care Medicine, EMGO Institute for Health and Care Research VU University Medical Center, Van der Boechorststraat 7, 1081 BT Amsterdam, The Netherlands

## Abstract

There is growing attention for chronic diseases in sub-Saharan Africa (SSA) and for bridges between the management of HIV/AIDS and other (noncommunicable) chronic diseases. This becomes more urgent with increasing numbers of people living with both HIV/AIDS and other chronic conditions. This paper discusses the commonalities between chronic diseases by reviewing models of care, focusing on the two most dominant ones, diabetes mellitus type 2 (DM2) and HIV/AIDS. We argue that in order to cope with care for HIV patients and diabetes patients, health systems in SSA need to adopt new strategies taking into account essential elements of chronic disease care. We developed a “chronic dimension framework,” which analyses the “disease dimension,” the “health provider dimension,” the patient or “person dimension,” and the “environment dimension” of chronic diseases. Applying this framework to HIV/AIDS and DM2 shows that it is useful to think about management of both in tandem, comparing care delivery platforms and self-management strategies. A literature review on care delivery models for diabetes and HIV/AIDS in SSA revealed potential elements for cross-fertilisation: rapid scale-up approaches through the public health approach by simplification and decentralisation; community involvement, peer support, and self-management strategies; and strengthening health services.

## 1. Introduction

There is growing attention for chronic life-long conditions (CLLCs) in sub-Saharan Africa (SSA) and for the challenge that these countries face in coping with rising numbers of patients with such diseases. The strong advocacy for managing noncommunicable diseases (NCD) appropriately, many of which are life-long, and the more general focus on health systems strengthening has catalysed attention for chronic care [1, 2]. This trend provides an opportunity to move away from the traditional divide between infectious and noninfectious diseases towards new frameworks for managing disease in a broader perspective [3, 4]. HIV/AIDS is the most eye-catching new chronic disease since antiretroviral treatment (ART) became available at large-scale. Despite this recognition, the models of care and approach for HIV/AIDS and other infectious diseases and for chronic NCD have historically grown separately and bringing these together is no easy task.

Health systems in SSA have developed with their major objective being the control of acute infections and improving maternal and child health. The rise of HIV/AIDS brought about a major change, because for the first time a chronic health problem received major attention, and called for the consideration of individual suffering and treatment. Delivery models for large-scale affordable ART led to the “public health approach”, a simplification and decentralisation of disease management to primary care level. Though still very much facility-based, this approach meant a breakthrough in thinking about health care delivery. The individual management of and care for NCD has been an almost exclusively High Income Countries' (HIC) affair until very recently; care models for diabetes mellitus and other chronic diseases were developed for these countries and included a strong emphasis on self-management and patient empowerment. However, these models are difficult to export to low-resource contexts, because of their focus on individual care and reliance on multidisciplinary teams and collaboration between primary and secondary health care institutions [5]. 

Estimates are that at present, 285 million people are living with diabetes, 33 million with HIV/AIDS, and 972 million with hypertension or cardiovascular diseases, with more than 50% of all of these in Low Income Countries (LIC) [6–9]. Projections for 2030 show that HIV/AIDS, ischemic heart disease and diabetes mellitus will be the 1st, 3rd, and 11th cause of DALYs lost [10]. The growing numbers of patients with Chronic Life-Long Conditions put an immense burden on health systems and populations, because of increased needs for health care providers and steadily rising costs of health care services. Although accurate estimates for chronic-illness health care expenditure in SSA are difficult to find, figures from high income countries show the unsustainability of the traditional professionalised models of chronic care: the direct health care cost for people with chronic conditions in the United States accounts for three quarters of the national health care expenditure while the cost of diabetes care in European countries is between 2 and 15% of national health expenditure [5, 11]. Household surveys from low income countries illustrate that chronic diseases are an important determinant for households facing catastropic health care expenditures [12, 13].

Although the literature increasingly identifies the potential bridges between HIV/AIDS and other chronic conditions' management, mentioning the need for self-management, and primary health care approach, authors do not elaborate further [14–16]. Little effort has been made to bring care models for infectious and noninfectious chronic diseases closer to each other. This separation of care models is increasingly becoming an anachronism with growing numbers of people living with both HIV/AIDS and other chronic conditions related to accelerated aging processes [17]. 

In this paper, we aim to address this gap, by looking at commonalities between diseases from the chronicity perspective and by reviewing models of care for these diseases. We focus on diabetes mellitus type 2 (DM2) and HIV/AIDS, because these, individually and in combination, are becoming increasingly important in SSA. We argue that in order to cope with care for HIV patients and diabetes patients, health systems in SSA need to adopt new strategies taking into account essential elements of chronic disease care. 

The paper consists of three parts. In the first part, we present a framework to describe differences and parallels between chronic diseases, looking at four disease-related dimensions and we apply this framework to compare DM2 and HIV/AIDS. What are the consequences for the organisation of care? What are potential bridges when it comes to care organisation/health system organisation for those diseases? In the second part, we review the present and potentially relevant practice of care for these two diseases in SSA, analysing existing models. What are the factors of success? What is the experience in practice? In the last part, we discuss the possibilities of cross-fertilisation and improvement of care for diabetes and HIV chronic care in SSA.

## 2. Methods

We performed our study in three phases. In the first phase, we developed a conceptual “chronic dimension framework” that allows the analysis of dimensions of chronic diseases relevant for the management and organisation of care. The framework was constructed through literature research and expert consultation. We started from the three groups of actors identified in the Innovative Care for Chronic Conditions (ICCC) framework (health care organisation, community, patient, and family) and a discussion among experts about the characteristics of chronic conditions [16]. During a 3-day workshop on health systems and chronic diseases with public health scientists, these dimensions were elaborated [18]. Further reading deepened the elements of each dimension. We then applied the framework to HIV/AIDS and diabetes, to explore commonalities and differences. 

The second phase comprised a literature review on care delivery models for diabetes and HIV/AIDS, according to the “decision support approach”, to develop a theory [19, 20]. Our search was guided by a number of questions. What models of care delivery and treatment of diabetes or HIV/AIDS patients are used to reach large-scale coverage in SSA? What are the channels of delivery, which kind of cadre is involved, and how are the community and patients involved? We reviewed two major databases (Pubmed and Google Scholar) and the websites of relevant organisations (UNAIDS, WHO and ministries of health of SSA countries). For HIV/AIDS, the search terms were “HIV”, “AIDS”, “ART”, “health care delivery”, “health care model”, “community health services”, “decentralisation”, and “peer support”, in combination with SSA. The search terms for DM were “diabetes”, “chronic care”, “chronic disease management”, “chronic care model”, “diabetes”, also in combination with “SSA” and specific country names. We selected those articles that focused on the description of delivery models, at conceptual or operational level, and distilled the most important elements of the existing models of care for both DM2 and HIV/AIDS.

In the third phase, we integrated the lessons from the review into the framework. We analysed whether the elements of existing care models for one disease would also be relevant for the organisation of care and management of the other one. Our recommendations were derived from a consensus procedure with the authors as participants.

## 3. A Framework for Chronicity 

The prevailing organisational design of health care is guided by health professionals and determined by the biomedical characteristics of a disease and medical care required. Is it infectious? How frequently are medical check-ups desirable? Which medical equipment should be available? However, chronic diseases result in a “biological disruption” for patients, meaning that experiencing a chronic illness in everyday life has enormous impact and necessitates a process to incorporate such illness into life and identity, in terms of cognitive processes and practical response [5]. In this process, the involvement of family and environment is indispensable. Recognizing this aspect means that optimal management of chronic diseases implies the adaptation of health care organisation to take into account this patient and environment perspective.

**Figure 1:**
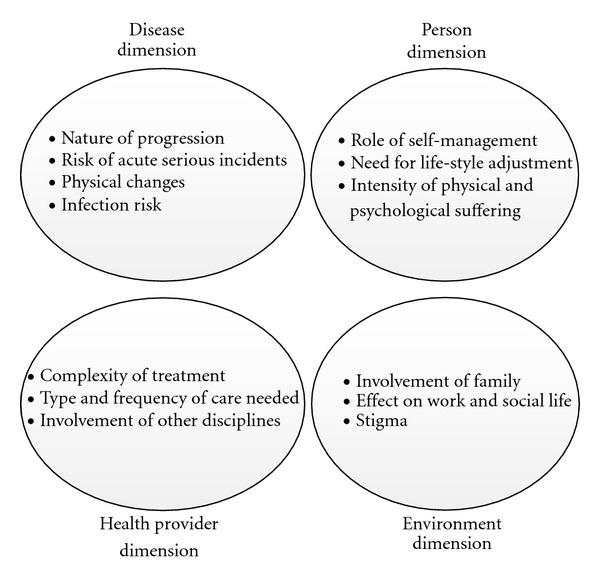
The “Chronic Dimension Framework” to describe four dimensions of chronic conditions.

We designed a framework to analyse these four dimensions of chronic diseases: the biomedical or “disease dimension”, the “health provider dimension”, the patient or “person dimension” and the “environment dimension” (Figure 1).

This “Chronic Dimension Framework” can be used to analyse the characteristics of any CLLC, to be able to identify and clarify the needs for management and support taking into account the disease-inherent characteristics and the perspectives of actors involved. Although all dimensions are related to each other and influence each other, the model emphasises the views of each actor (patient, provider and people in the environment) as key variables on their own, not necessarily congruent with each other [21]. We will shortly elaborate on them.

**Figure 2:**
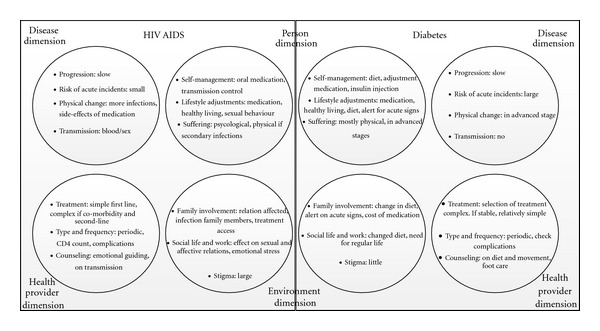
Comparing the chronicity dimensions of HIV AIDS and diabetes.

The disease dimension refers to the biomedical characteristics, inherent to a disease. Examples are the onset and the nature of progression-for instance the pace, level of (un)certainty and interpatient variability, the risk for acute fatal incidents, the physical changes, the risk of infection, and mode of transmission [22]. The health provider dimension entails the professional involvement with patients and their disease. It raises questions such as: how complex is the treatment; who can provide the treatment; how often is patient contact necessary and what does it entail? What is the provider's point of view towards the patient and his/her disease [21]? Are professionals of other disciplines involved? The person dimension involves the experience of patients themselves, their attitude towards their disease, their way of coping, and their own role in disease management. What is the role of self-management? What sort of life-style adjustments are desirable and possible? How much do patients suffer, physically and/or psychologically [23]? The environment dimension relates to all actors (both personal and institutional) interacting with patients and potentially influencing their way of coping and their disease management. This starts from the inner circle of family and household members, but extends to the consequences for work and social life and to the role of stigma and how people feel depicted in society. We applied this framework to compare two increasingly prominent CLLCs in SSA, HIV/AIDS and DM2 (Figure 2). 

The disease dimension of DM2 and HIV/AIDS is that of a slowly progressive disease, which in advanced stages leads to increased morbidity affecting multiple organ systems, requiring different professional expertise. The threat of exposure to opportunistic infections such as meningitis and tuberculosis, especially in SSA, contributes to uncertainty about the progression of HIV/AIDS and a more pronounced premature mortality for HIV/AIDS than for diabetes, especially in absence of ART. DM2 usually shows a gradual progression, but entails a considerable risk of acute life-threatening incidents such as hypo- and hyperglycaemia. The most striking difference is the mode of transmission. Diabetes is a noncommunicable disease, with a hereditary component, triggered by life-style factors such as diet and physical exercise. HIV/AIDS is infectious and is transmitted through blood or venereal contact, its transmission being associated with behaviour and lifestyle aspects that increase the chance of infection. This diversity in risk factors and transmission necessitates different strategies at population and individual level to control the spread of disease. The risk of infection has a large impact on how the patient and his environment interact, which we will discuss under these dimensions.

From the health provider dimension, HIV/AIDS and DM2 are managed quite differently. Nowadays, first line treatment for HIV/AIDS is straightforward, comprising one or two tablets a day, although patients side-effects are still an important barrier to treatment adherence [24]. Diabetes treatment is more complicated, in terms of the choice of treatment regimen, the combination of diet, tablets and sometimes insulin and the adjustment of treatment to variation in diet and exercise. Diabetes treatment can be standardised into flowcharts, but these involve multiple steps in decision-making and require some expertise and training to handle them correctly [25]. The risk for an acute life-threatening incident requires the availability of 24-hour medical advice. These differences influence the feasibility of decentralising treatment beyond primary care level. For both DM2 and HIV/AIDS, the routine follow-up is periodic and involves mainly monitoring of treatment and disease progression. The crucial role of the professional health provider is to detect and manage complications. The overall management of both groups of patients entails, besides the medical tasks, a lot of counselling on how to live with the disease. 

The person dimension is highly affected for both DM2 and HIV/AIDS, but in quite different ways. DM2 requires a lot of adjustments in the social spheres of life, such as changing diet, maintaining a daily routine, and reducing other cardiovascular risk factors such as smoking. People living with HIV/AIDS (PLWHA) might face less intrusive lifestyle adjustments, as they are merely related to avoiding risk behaviour, but they generally do experience a large psychological set-back from the diagnosis and its consequences. Whereas diabetes is considered a disease you deserve no blame for (and in some contexts even seen as a sign of wealth), PLWHA are confronted with stigma and this often leads to a great deal of psychological suffering. This stigma has many origins, like the association of infection with sexual contact in general, with particular behaviours considered risky and stigmatised in themselves (homosexuality, drug addiction, prostitution, or promiscuity), but also the lack of information or the fear for a potentially lethal disease [26]. This stigma, the change in attitude of people towards PLHA, the ideas of PLHA themselves, and the real risk of infecting others with a potentially lethal disease leads to a lot of psychological distress and to an (implicit or explicit) barrier between the person affected and his/her environment [27, 28]. This barrier hinders the search for and finding of social and health service support [29], and the result is that often necessary support is not found. Physical suffering usually comes in advanced stages for both groups of patients. The role of self-management in DM2 comprises life-style adjustments discussed above, monitoring of glucose levels and adaptation of medication, and recognition and management of acute danger signs. The “technical” tasks of self-management of HIV/AIDS are less complicated, focusing on intake of oral medication, coping with side-effects, and knowing what to do in case of interruption, and on the control of transmission. Whereas people with diabetes observe an immediate influence of their behaviour and subsequent glucose levels on their well-being, the signs of not taking medication for PLWHA arise more gradually but can be more ominous on the longer term, because of the development of virus resistance. 

The environment dimension for people with DM2 and those with HIV/AIDS is greatly affected, especially the immediate circle of family life. Family members of DM2 patients encounter the changes in meal patterns and composition which usually affect the whole family or require the cooking of different meals, but they also learn to recognize acute signs of hypo-/hyperglycaemia and other complication symptoms. HIV/AIDS affects the sexual and affective relationships between partners, but possibly also with children. In families of both patient groups, concerns about access to treatment can have a large impact on the routine activities and other needs in the family. In a similar way, social life and work can be affected by the disease. As discussed above, the environment's perception of HIV/AIDS and DM2 is very different. The stigma of HIV/AIDS makes it difficult for PLWHA to share with others, but it can bring patients closer to each other [30]. The still dominant perception of diabetes as a disease for the rich leads to a more individualised experience.

Our framework reveals that HIV/AIDS and DM2, at first sight very different diseases, also share some comparable characteristics in the health provider, person, and environment dimensions. The health provider dimension is crucial for the organisation of care, for example, the choice of the most appropriate platforms for delivering medical care, the need for a permanent service within reach, the involvement of other professionals, the possibilities for self-management, and the role of families. The person and environment dimensions determine the kind of adjustments, feasibility, and challenges when empowering patients and involving the family and others in helping the patient to manage his/her disease. Although the content of life-style adjustment and impact might be different, the experienced intensity of the disease, the processes involved in motivation, empowerment, and behavioural changes are similar. The comparison illustrates that it is useful to think about management of diabetes and HIV/AIDS in health systems in tandem, to compare care delivery platforms, strategies for self-management, and involvement of the environment. In the following section, we will look at the practice of care for both diseases, to see which lessons can be learnt.

## 4. Present Models of Care for Diabetes and ****HIV AIDS for SSA

For both diseases, there is not one care model, such as the “DOTS approach”, which has been universally endorsed by the WHO for tackling tuberculosis [31]. Instead, many organisations have experimented with an approach of their own, leading to various delivery models. Some of them have been endorsed at national level and subsequently scaled up.

Many early publications about ART in SSA address the barriers to long-term quality care, such as costs, interrupted drug supplies, the lack of referral system, weak clinical management with insufficient attention for retention in care, and provider-patient interaction and poor social support [32]. Some early care projects for PLHA were modelled upon the DOTS strategy, but the approach was doubted to be useful and appropriate for a Chronic Life-Long Condition such as HIV/AIDS [33–35]. The Millennium Development Declaration turned the HIV/AIDS epidemic into a matter of international political urgency and increased resources facilitated programmes to deliver treatment and care to PLWHA [36]. Access to ART in low- and middle income countries increased from 400 000 in 2003 to 6.65 million in 2010, or 47% coverage of people eligible for treatment [37]. Many ministries, international development actors, and local organisations experimented with delivery models, while also trying to expand coverage at the same time. 

There are only a handful of publications on small-scale experiments to provide care for DM2 in SSA. The 2011 NCD summit resulted in five priorities for national strategies, but the care component has so far received only minimal attention, mainly mentioning the need for a primary care based delivery and access to essential medicine [38]. The reality in SSA is that care for people with diabetes is of low quality. Routine practice is that care in the public sector is mostly provided at secondary level and that the gap at the first line is filled by a variety of private providers. 

### 4.1. HIV/AIDS Care

The papers we retrieved from our review included programmatic guidelines of the World Health Organisation (WHO) and of Ministries of Health of SSA countries; papers describing individual (single or multi-country) studies with various models of care; systematic reviews; and more general articles identifying barriers to care and health system links. We found papers at pilot project level from Tanzania, Malawi, South Africa, Zambia, and Malawi [39–44]. The delivery approaches described were mostly centred at primary care level with a strong community component sometimes using additional tools, such as mobile phones. From reviewing these papers, we distilled three major issues, which we elaborate further: (1) rapid scale-up approaches through the public health approach; (2) community and peer support; and (3) strengthening the health services in which care is embedded.

**Rapid Scale-Up Strategy through the Public Health Approach**

To rapidly scale-up ART in SSA, where health systems are overall weak and have huge shortages of health workforce, it was soon realised that the care model from Europe and the United States with an individualised medical approach, hospital-based clinics, specialist consultation, and regular follow-up of clinical parameters was not possible. To deal with these challenges, WHO proposed a “public health approach” which prioritised large-scale access to treatment over maximising individual treatment. The main principles of the public health approach are simplification of treatment protocols and clinical monitoring; decentralisation of ART care delivery to the local health centre and community level; and task shifting and involvement of community and PLWHA in program design, management, and care [45].

Diagnostic and treatment protocols were rationalised and standardised, by reducing the number of laboratory tests and introducing Fixed Dose Combinations (FDC) tablets. Core responsibilities and core tasks were delegated to lower cadre health workers [40, 46, 47]. For instance, nurses were trained and became responsible to initiate and follow up patients on first line ART [40, 48]. New health cadres were created to diminish the workload in health facilities and to reinforce the link with the community, for instance lay providers who could provide specific ART care delivery functions like adherence support, defaulter tracing, education, and counselling [49]. The results of these approaches indicate that they can successfully improve access to ART with good quality. Pilot projects in Tanzania, Malawi, South Africa, Zambia, and Mozambique show similar or improved treatment outcomes for PLWHA receiving decentralised care compared to hospital-based care [44, 46, 50–54].

**Community and Peer Support**

In many HIV/AIDS programmes, there is a large role for the community and for patient groups. These community and peer supports usually take up tasks like psychosocial or adherence support and defaulter tracing [55, 56]. The main results published relate to improved retention of patients in care, when such persons are involved in care delivery [57–61]. Community and patient involvement can contribute to their empowerment. To ensure lifelong adherence to treatment, it is important to involve PLWHA in their daily care and to demedicalise care where possible, for instance by separating medical consultation from drug refills. Some pilot projects show the feasibility to involve the community and PLWHA also in medical tasks such as ART provision in the community in Mozambique and Kenya [56, 62, 63]. The main pillars of these projects are the empowerment of the PLWHA, peer support, and information sharing.

**Strengthening Health Services and Health Systems**

Conditions for successful decentralisation to primary care level are that such facilities function well, that a minimum of required support services are in place (for instance referral laboratory, trained human resources, continuous drug supply) and that other essential functions are not endangered by the additional tasks of ART. This led to the realisation that decentralisation and scaling-up of ART also necessitated investment in workforce and in the general infrastructure [64–66]. In two countries with successful scaling-up, Malawi and Ethiopia, HIV earmarked donor funding was used to strengthen the health system. The expansion of health workforce contributed to an overall increase in functional health facilities and an improvement of utilization rate and health outcomes was noticed [67].

### 4.2. Diabetes Care

There are very few publications about delivery of diabetes care in SSA, most of them describing local-level initiatives, without explicit reference to a framework. We also included papers which elaborate models often referred to as examples for SSA [11]. The papers reviewed included descriptions of models for care for chronic diseases and their meta-evaluations; specific models of care for diabetes; overviews of diabetes care in SSA or particular countries; individual studies on delivery of care. The publications about diabetes care in SSA came from South Africa [68], Ethiopia [69, 70], DR Congo [71], Tanzania, and Cameroon [72, 73].

The most known model is the Chronic Care Model (CCM), which has been implemented and evaluated in many high income countries [74]. It proposes a redesign of care organisation, to include self-management; clinical guidelines; an information system allowing for stratification according to risk profiles and subsequent follow-up; reorganising care delivery focusing on teamwork and continuous care; creating community linkages and mobilising resources [75]. Meta-analyses indicate that implementation of the CCM improves quality of care and clinical outcomes, but there is no clarity about which elements are essential or to what extent [76, 77]. There have been initial steps to introduce the CCM in Uganda and in Ethiopia, but these have not yet been evaluated [2, 78]. Although not explicit, some chronic disease projects in SSA bear features of the CCM, particularly clinical guidelines, education programmes, and continuous care [72]. The WHO Innovative Care for Chronic Conditions (ICCC) Framework is an adaptation of the CCM, which expands to the policy environment and puts more emphasis on the role of the community, to be applicable also in LIC [16]. The ICCC framework has only incidentally been used in practice and has, as far as we know, not been implemented at operational level in SSA [79]. In 2011, the WHO published a Package of Essential Noncommunicable (PEN) Disease Interventions for primary health care in low resource settings, which does not entail a real “model” for delivery, but provides a number of tools to organise care for a noncommunicable disease [80].

From the experiences with models in HIC and from the publications about projects in SSA, we have identified two important issues: (1) decentralisation and task-shifting; and (2) involvement of patient groups and self-management strategies.

**Decentralisation and Task-Shifting**

Most of the projects in SSA started from a hospital and were then extended to the local health centre level, executed by nurses, and supervised by mobile (teams of) specialists. Training, supervision and diagnostic and treatment protocols were developed to support health centre staff [68–72]. Those projects which reported on results, mention that people remain in follow-up and that clinical outcomes are comparable with specialist care. Approximately 80% of all patients in the district could be treated at primary care level [68, 70]. Similar to the ART approach, decentralisation processes entailed task-shifting to lower cadre, differentiation between routine and complicated cases, training of staff and education to patients and families, affordable drugs supply, and sometimes additional social care for the most vulnerable groups.

**Involvement of Patient Peer Groups and Self-Management Strategies**

Self-management is a CCM component, but has also been developed and implemented as a strategy in itself, in order to support patients with chronic diseases to develop knowledge and skills necessary for self-care [81, 82]. Self-management strategies can improve clinical outcomes and patient empowerment [83–86]. Self-management and peer support are reciprocal dimensions that are often combined in support programmes. Peer patient groups learn about how to cope with and self-manage their disease and peer groups develop mechanisms to support each other, materially, mentally, and socially [83, 87].

For diabetes, self-management has been translated into an education programme focusing on problem-solving, decision-making, and confidence-building of patients, addressing diabetes-specific dimensions [88, 89]. Peer support for diabetes has been described in LIC outside of SSA, such as Cambodia [90]. In SSA, there is a growth of national and local diabetes patient associations, for instance to organise access to treatment or to stimulate peer support and there is increased attention for their potential [5, 73, 91, 92]. 

## 5. Lessons to Improve Chronic Care

**Figure 3:**
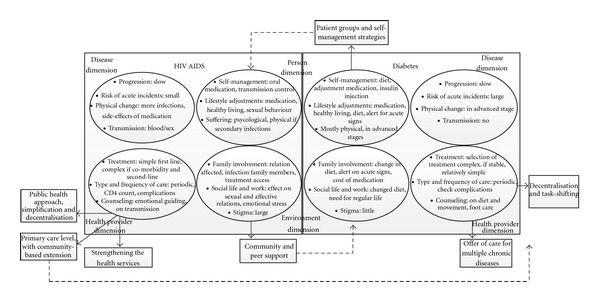
Potential cross-fertilisation of present care models of HIV/AIDS and diabetes, taken into account the chronicity dimensions.

In the former section, we distilled lessons from the present models of care for HIV/AIDS and DM2, relevant for SSA. An inclusion of these lessons into our chronic dimension framework shows the potential for cross-fertilisation between models (Figure 3). In order to cope with chronic care for PLWHA and people with diabetes, health systems in SSA need to move to simplification of treatment and encouragement of self-management.

Although there are differences in complexity of treatment, practice shows that decentralisation and task-shifting is possible for both DM2 and HIV/AIDS. The public health approach, focusing on rapid scale up and simplification and standardisation of treatment, could be much more exploited to improve access to diabetes treatment. The treatment guidelines for SSA of the World Diabetes Federation are a useful document in this sense, but they are not very widely distributed and used [25]. The FDC tablet meant huge progress in the simplification of ART, making it much easier for PLHA to adhere to treatment. There are also gains to be made in the access to and rational use of blood glucose testing materials, insulin, and oral antidiabetic medication in LIC [93, 94]. A universal minimal monitoring package would greatly facilitate simplification and comparison [95]. However, the large and growing number of patients living with one or both diseases makes it urgent to transfer more responsibilities to patients themselves [5]. Argumentation goes beyond rationalisation of resources and should be part of strategies to empower patients to cope with their CLLC [5]. HIV/AIDS models have a lot of experience with activating community and peer support for patients, but the concept of self-management has been hardly discussed in the scope of individual care. The experiments that involve PLWHA in the delivery of ART are promising and should be further evaluated. The patient associations for HIV/AIDS and diabetes have large potential, but their objectives and strategies are often not yet well-developed. Both groups could learn a lot from self-management programmes developed in HIC.

The last lessons from our analysis relate to health system organisation. The HIV/AIDS models illustrate that decentralisation to primary care level cannot be realised without strengthening the primary care services themselves. This means for instance investment in laboratory analysis, patient centred care, and counselling services and follow-up. This could benefit the primary care for other chronic diseases. Some of the projects discussed expanded their care model to other chronic conditions, for instance from diabetes towards also hypertension and asthma patients [68, 72]. A more in-depth evaluation of such an integrated project which also includes HIV/AIDS patients has been described in Cambodia, which showed that “staff could effectively assume a multidisciplinary role and that skills to manage patients who need to start lifelong treatment were relevant to and effective for both HIV/AIDS and diabetic care”, for instance a patient-centred approach and adherence support [96]. The bundled care for diabetes and other chronic diseases is particularly relevant for DM2 and HIV/AIDS, since both diseases and their combination are increasingly important in SSA, because ART-induced diabetes leads to increased comorbidity, and because the rising incidence of diabetes in SSA results also in more patients who happen to have both diseases. However, comorbidity of chronic diseases is a quantitatively important phenomenon, in general practice among elderly people, but even more prominent and at a younger age among PLHA [17, 97]. Therefore, the lessons from our paper are also relevant for other CLLC, such as hypertension and chronic lung disease.
